# Electrochemical Sensor Based on Glassy-Carbon Electrode Modified with Dual-Ligand EC-MOFs Supported on rGO for BPA

**DOI:** 10.3390/bios12060367

**Published:** 2022-05-27

**Authors:** Rui-Hong Ye, Jin-Yang Chen, Di-Hui Huang, Yan-Jun Wang, Sheng Chen

**Affiliations:** 1 Fujian Province-Indonesia Marine Food Joint Research and Development Center, Fujian Polytechnic Normal University, Fuqing 350300, China; ruihye@163.com (R.-H.Y.); wang0455@fpnu.edu.cn (Y.-J.W.); chensheng@fjnu.edu.cn (S.C.); 2 College of Life Sciences, Fujian Normal University, Fuzhou 350117, China; chenjyen@163.com

**Keywords:** electronic conductive metal-organic frameworks, dual–ligand MOFs, reduced graphene oxide, bisphenol A, electrochemical sensor

## Abstract

The electronic conductive metal-organic frameworks (EC-MOFs) based on a single ligand are not suitable for the accurate detection of bisphenol A (BPA) due to the limitations of their electron-transfer-based sensing mechanism. To overcome this drawback, we developed EC-MOFs with novel dual-ligands, 2,3,6,7,10,11-hexahydroxy-sanya-phenyl (HHTP) and tetrahydroxy 1,4-quinone (THQ), and metal ions. A new class of 2D π-conjugation-based EC-MOFs (M-(HHTP)(THQ)) was synthesized by a self-assemble technique. Its best member (Cu-(HHTP)(THQ)) was selected and combined with reduced graphene (rGO) to form a Cu-(HHTP)(THQ)@rGO composite, which was thoroughly characterized by X-ray diffraction, field scanning electron microscopy, and energy-dispersive X-ray spectroscopy. Cu-(HHTP)(THQ)@rGO was drop-cast onto a glassy carbon electrode (GCE) to obtain a sensor for BPA detection. Cyclic voltammetry and electrochemical impedance tests were used to evaluate the electrode performance. The oxidation current of BPA on the Cu-(HHTP)(THQ)@rGO/GCE was substantially higher than on unmodified GCE, which could be explained by a synergy between Cu-(HHTP)(THQ) (which provided sensing and adsorption) and rGO (which provided fast electron conductivity and high surface area). Cu-(HHTP)(THQ)@rGO/GCE exhibited a linear detection range for 0.05–100 μmol·L^−1^ of BPA with 3.6 nmol·L^−1^ (S/N = 3) detection limit. We believe that our novel electrode and BPA sensing method extends the application perspectives of EC-MOFs in the electrocatalysis and sensing fields.

## 1. Introduction

Porous molecular materials, also known as metal-organic frameworks (MOFs) or porous coordination polymers (PCPs) [1], attract a lot of interest from scientists and engineers as valuable materials for applications related to gas adsorption and separation [2], sensing [3], catalysis [4], energy storage [5], etc. Most MOFs are non-conductive and insulators, which limits their broader usage in electrochemical applications [6]. However, a recent discovery of electronically conductive MOFs (EC-MOFs), containing periodically connection cations and electrochemically active organic units [7], allowed scientists and engineers to overcome this drawback and to synthesize dozens of EC-MOFs with permanent porosity and conductivity and useful for semiconductor-based devices [8], electrocatalysts [9], supercapacitors [10], chemiresistive gas sensors [11], etc.

Two-dimensional (2D) π-conjugated EC-MOFs also possess semiconducting properties and good chemical stability [12]. However, these EC-MOFs contain only a single redox-active organic ligand [8], which makes it hard to adjust and tune the conductivity and topology of these compounds to satisfy the need of some electronic applications.

This drawback could be overcome by using MX_2_Y_2_-based EC-MOFs, where M is a metal cation, and X and Y could be N, S, or O but cannot be the same element. Usage of these MOFs not only expands their variety and applicability to electronic fields but also allows scientists to engineer the built-in structural tunability in these 2D MOFs. Thus, Jiang et al. [13] synthesized 2D MX_2_Y_2_-type copper 1,3,5-triamino-2,4,6-benzenetriol metal-organic framework (Cu_3_(TABTO)_2_-MOF) and used it to understand the role of O_2_ in the synthesis of MOF-containing redox-active ligands. Wu et al. [14] demonstrated that 2,3,6,7,10,11-heximinotriphenyl (HITP)-doped Cu-HHTP high-quality nanofilms can accurately adjust chemical resistance and, as a result, sensitivity and selectivity towards benzene. Yao et al. [15] developed a new 2D π conjugated EC-MOF, Cu_3_(HHTP)(THQ), and demonstrated that its resistance and active site accessibility could be changed by adjusting its conductivity and porosity. They then built an excellent gas sensor based on these properties.

Bisphenol A (BPA) is an endocrine disruptor, mimicking the estrogen effects. In the past, it was a component of plastics widely used to fabricate baby bottles [16], food cans [17], beverage containers [18], etc. However, it was later shown that even trace BPA contents could cause brain damage, immune system failure, thyroid, and other diseases [19,20]. Even though BPA-containing plastics are now banned, it is still present in the environment due to the discarded material. Thus, scientists are still concerned about the harmful effects of BPA and seeking a reliable and rapid method for its detection. 

Current BPA detection techniques are bulky, complex, and expensive setups (e.g., high-performance liquid chromatography-mass spectrometry (HPLC-MS) [21], fluorescence spectroscopy [22], enzyme-linked immunosorbent assay (ELISA) [23], chemiluminescence immunoassay [24], etc.). Most of these methods also require multi-step sample pretreatment. Recently, electrochemistry-based detection methods started to attract scientists as they offer fast responses, high sensitivity, low cost, and simplicity [25], as well as environmental friendliness and almost no sample pretreatment. Electrochemical methods based on modified electrodes are especially attractive because they demonstrate significantly higher peak currents [26,27,28]. Modifications to glassy carbon electrodes (GCEs, which typically demonstrate good resistance and stability), are especially popular because of the GCE’s somewhat slow surface electron transfer rate and a need for high overpotentials to induce the redox reactions [29,30]. BPA detection with MOFs sensors has been extensively evaluated [31,32]. However, suitable candidate materials are still needed for the realisation of a BPA-sensing platform with an excellent selectivity, high response rate and low detection limit. Moreover, a BPA electrochemical sensor of dual-ligand EC-MOFs has not yet been reported. 

One of the popular GCE modifications is with graphene-based materials. Graphene possesses a very high surface area (up to 2630 m^2^/g) and a 2D layered structure [33]. Graphene oxide (GO) also offers numerous epoxy, hydroxyl (OH) and carboxyl surface functional groups [34], capable of adsorbing other compounds and also useful for targeted surface functionalization [35,36] and other complex molecules attachment (including MOFs). GO could be reduced to rGO, which restores some of the C=C bonds and increases the overall material’s conductivity [37,38,39]. 

This paper reports a GCE modified with a double-ligand EC-MOFs, Cu-(HHTP)(THQ) (abbreviated as Cu-H-T) and reduced graphene oxide (rGO), which was then used for BPA detection. First, we synthesized a series of EC-MOFs, M-(HHTP)(THQ) (abbreviated as M-H-T), to screen compounds with the best BPA detection performance. The best M-H-T (which was Cu-H-T) was then further modified with rGO to take advantage of its high charge transfer rate, beneficial for increasing electron transfer between analyte and electrode. Our novel Cu-H-T@rGO/GCE was tested for voltammetric BPA detection in beverage bottles and showed an outstanding performance. 

## 2. Experimental

### 2.1. Materials

Tetrahydrochloride (FeCl_2_∙4H_2_O), cobalt hexachloride (CoCl_2_∙6H_2_O), nickel (II) hydrated acetate (NiC_4_H_6_O_4_·4H_2_O), copper pentahydrate sulfuric acid (CuSO_4_∙5H_2_O), hexahydrate zinc nitrate (Zn(NO_3_)_2_∙6H_2_O), 2,3,6,7,10,11-hexahydroxytriphenylene (HHTP), ethylenediamine, ethanol (EtOH), acetone (CH_3_COCH_3_), N,N-Dimethylformamide(DMF), BPA, 4-tert-Octylphenol(POP), 4-Nitrophenol(PNP), and Tetra-bromo-bisphenol A (TBBPA) were purchased from Aladdin Industrial Co. Tetrahydroxy-1,4-quinone (THQ) was received from Energy Chemical Co. (Shanghai, China). rGO was supplied by Nanjing Xianfeng Nanomaterials Technology Co. (Nanjing, China) It was physically reduced and its specific surface area is 500–1000 m^2^/g. Nafion was purchased from Alfa Aesar (Shanghai, China). All other reagents used were analytical grade and used directly without any purification. Double distilled water (with conductivity above 18 MΩ) was used throughout this work. All experiments were conducted at 25 ± 2 °C.

### 2.2. Instrumentation

Field emission scanning electron microscopy (FE-SEM, Nova Nano SEM 230, FEI, Hillsboro, OR, USA) and powder X-ray diffraction (XRD, Ultima IV, Rigaku, Tokyo, Japan) were used to characterize the morphology and microstructure of the modified GCE. The electrochemical tests were carried out using an electrochemical CHI660E workstation manufactured by CH Instruments (Shanghai, China). For this purpose, we used a three-electrode system containing bare or modified GCEs (3 mm in diameter), Pt wire as an auxiliary electrode, and a saturated (with 3 mol·L^−1^ KCl) calomel electrode (SCE, Shanghai, China) as a reference. All potentials were calculated relative to SCE. Buffer solutions based on phosphates (PBS, Laboratory homebrew) were used as electrolytes.

### 2.3. The Synthesis of Double-Ligand EC-MOF M-H-T and Cu-H-T@rGO Nanocomposite

**Scheme 1:**
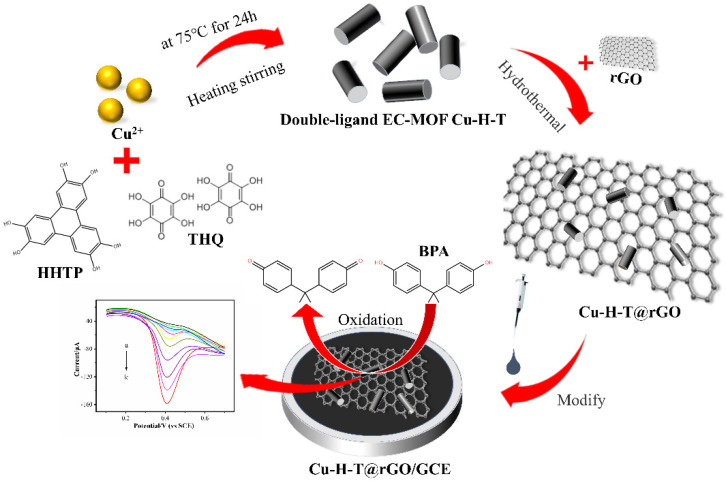
Synthesis of double-ligand EC-MOF Cu-H-T@rGO/GCE.

The double-ligand EC-MOF Cu-H-T synthesis was based on a procedure described elsewhere [15,40]. For this purpose, as shown in Scheme 1, 5 mL of aqueous solutions A (containing 0.1 mmol of CuSO_4_∙5H_2_O and 11.5 μL of ethylenediamine) and B (containing 0.02 mmol of HHTP and 0.02 mmol of THQ) were ultrasonically mixed for 15 min and then transferred into a 20 mL test tube. In addition, 5 mL of the upper solution was then removed and replaced by 5 mL of fresh solution A. The test tube was then sealed and kept at 75 °C for 24 h. The black precipitate was rinsed with water, separated by centrifugation at 4000 rpm for 10 min and rinsed again. The solids were filtered, washed with H_2_O and acetone, and vacuum-dried at 65 °C overnight. Single ligand EC-MOF Cu-H polyhedrons were also prepared but without THQ addition.

We also prepared samples with different CuSO_4_∙5H_2_O amounts and also various metal ions (by replacing CuSO_4_∙5H_2_O with FeCl_2_∙4H_2_O, CoCl_2_ 6H_2_O, NiC_4_H_6_O_4_·4H_2_O, or Zn(NO_3_)_2_∙6H_2_O to obtain Fe-H-T, Co-H-T, Ni-H-T, and Zn-H-T, respectively) using the same procedure described above). 

Double-ligand EC-MOFs@rGO were also synthesized hydrothermally. For this purpose, rGO suspension (10 mg in 20 mL of water) was mixed with 20 mL Cu-H-T solution (in 0.1 mol∙L^−1^ NaOH), stirred for 2 h and then placed into a hydrothermal reactor for 6 h at 120 °C. The resulting product was centrifuged with 300 mL of deionized water and 300 mL of ethanol several times and vacuum dried at 60 °C for 12 h. Single ligand Cu-H@rGO composite was prepared following the same procedure but using only one ligand compound. 

### 2.4. Electrochemical Measurements

GCE was polished with Al_2_O_3_, ultrasonicated in a water/ethanol mixture and treated electrochemically using 1 mol·L^−^^1^ molsulfuric acid. The GCE performance was then tested using cyclic voltammetry (CV) in 5 mmol·L^−1^ [Fe(CN)6]^3−/4−^ containing 0.1 mol·L^−1^ of KCl, after which the GCE pretreatment was considered completed. 

In addition, 5 mg of EC-MOFs prepared in this work was mixed with 5 mL of DMF containing 0.2% Nafion and sonicated for 40 min. Furthermore, 6 μL of the resulting homogeneous slurry was then applied to the pretreated GCE surface. The modified GCE was dried at 50 °C for 30 min. The electrodes were marked according to the EC-MOFs used for modification (for example, Cu-H-T@rGO/GCE).

CV tests of modified GCEs were performed at 100 mV/s scanning rate in the 0.2–0.8 V range. Electrochemical impedance spectroscopy (EIS) was performed in 5 mmol·L^−1^ [Fe(CN)_6_]^3−/4−^ containing 0.1 mol·L^−1^ KCl in the 1–10^5^ Hz range with ±5 mV of allowed disturbance. Differential pulse voltammetry (DPV) was conducted at 100 mV amplitude, 0.3 s cycle, 100 ms pulse duration, and 10 s intervals between the pulses. The detection limits (LODs) were calculated by testing the corresponding electrodes against 10 minimum concentration of BPA solution. The final LOD value was reported as an average, with the error calculated as a standard deviation of 10 measurements.

## 3. Results and Discussion

### 3.1. Selection of Double-Ligand EC-MOF M-H-T Materials and the Feasibility of the Cu-H-T@rGO/GCE Electrochemical Sensors

**Figure 1:**
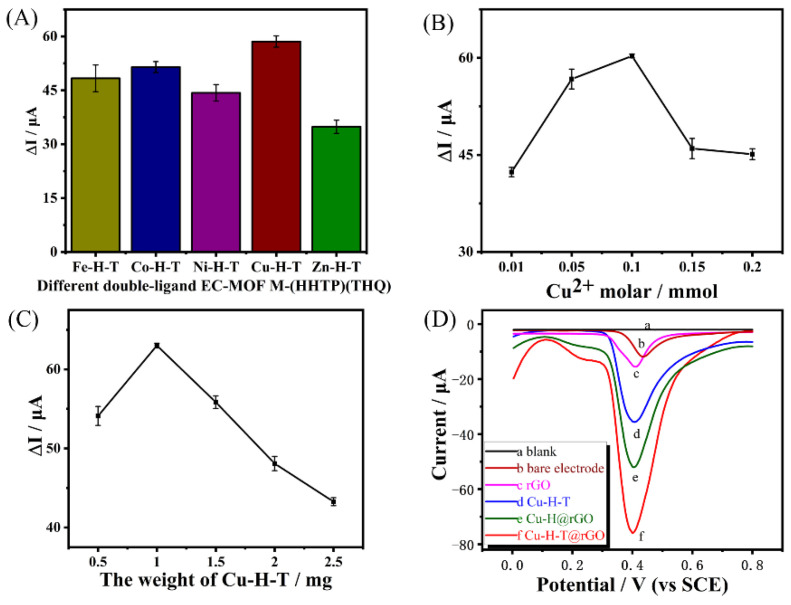
Current responses (**A**) of various M-H-T/GCEs, (**B**) of Cu-H-T@rGO/GCE containing Cu-H-Ts prepared using different Cu^2+^ contents (but constant HHTP and THQ contents, both equal to 0.02 mmol), and (**C**) of Cu-H-T@rGO/GCE containing different Cu-H-T/rGo ratios (expressed as Cu-H-T weight in the mixture) to a 0.1 mmol·L^−1^ BPA solution. Each data point in (**A**–**C**) represents an average of five measurements, while the error was calculated as a standard deviation. (**D**) DPV of blank, bare GCE, rGO/GCE, Cu-H-T/GCE, Cu-H@rGO/GCE and Cu-H-T@rGO/GCE under the presence of 0.1 mmol·L^−1^ BPA at pH = 7.0.

As shown in Figure 1A, the comparison of the oxidation current responses of GCEs modified with double-ligand EC-MOF-M-H-T materials (Fe-H-T, Co-H-T, Ni-H-T, Cu-H-T and Zn-H-T) towards 0.1 mmol·L^−1^ BPA revealed that the Cu-H-T/GCE demonstrated the highest peak current. This is probably because, compared with other metal ions, Cu ions in MOF have a stronger adsorption capacity for BPA [41,42]. Therefore, the conductive Cu-H-T was selected to synthesize Cu-H-T@rGO nanocomposites to be later used to prepare Cu-H-T@rGO/GCE.

To further optimize the electrochemical properties of Cu-H-T, we varied the Cu^2+^/ligands ratios by increasing Cu^2+^ initial content from 0.01 to 0.2 mol but keeping the HHTP and THQ amounts the same (both equal to 0.02 mmol). The responses of the corresponding Cu-H-T@rGO/GCEs towards BPA solution first increased but then decreased as a function of initial Cu^2+^ content (see Figure 1B). The highest oxidation current was obtained for the electrode prepared using 0.1 mmol Cu^2+^ solution. Thus, this Cu-H-T EC-MOF possessed the most catalytic site and the best conductivity, both of which were very beneficial for facilitating BPA adsorption and accelerated electron transfer. Therefore, Cu-H-T synthesized using 0.1 mmol Cu^2+^ solution was chosen for the next round of performance screening tests, which involved determining the optimum amount (by varying the initial CuSO_4_∙5H_2_O amounts from 0.5 to 2.5 mg) of Cu-H-T needed to obtain the best performing Cu-H-T@rGO/GCE. The highest peak oxide current under the presence of BPA was observed for the Cu-H-T@rGO/GCE prepared using 1 mg of CuSO_4_∙5H_2_O (Figure 1C). Such an excellent performance was obtained because Cu-H-T@rGO contained the best combination of active catalytic (adsorption) sites and the most optimum Cu-H-T/rGO ratio. Therefore, all consequent tests (including SEM, EDS, XRD and electrochemical characterization) were performed using Cu-H-T@rGO nanocomposites prepared using 1 mg of CuSO_4_∙5H_2_O.

DPV curves recorded for blank (no BPA added) buffer solution, bare GCE, rGO/GCE, Cu-H-T/GCE, Cu-H@rGO/GCE, and Cu-H-T@rGO/GCE under the BPA presence showed oxidation peaks (with the exception DPV performed in the blank buffer solution, see Figure 1D). The bare GCE showed the lowest oxidation peak due to the weak BPA adsorption on its surface. The rGO/GCE and Cu-H-T/GCE oxide currents were significantly higher, which was attributed to the higher number of conductive and BPA-adsorbing sites due to the presence of the rGO and Cu-H-T. Additionally, we confirmed that GCE modified with only rGO or only Cu-H-T could still detect BPA. The peak current of Cu-H@rGO/GCE increased even further, indicating a facilitated adsorption and charge transfer due to the synergetic interaction and tight interface between Cu-H and rGO. The highest catalytic activity and BPA oxidation current peaks were observed for the Cu-H-T@rGO/GCE, even at low voltages. Besides synergetic interaction between Cu-H-T and rGO, enhanced microporosity and available active centers of Cu-H-T contributed to such excellent performance.

### 3.2. Characterization of Modified Electrode Material

**Figure 2:**
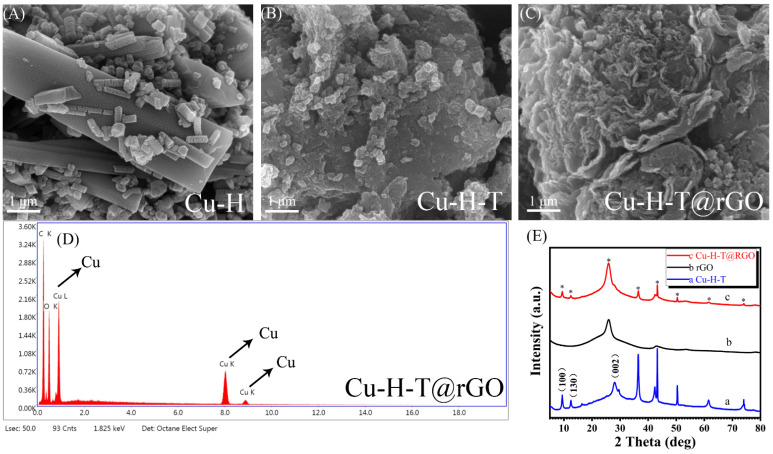
FESEM micrographs of Cu-H (**A**), Cu-H-T (**B**), and Cu-H-T@rGO (**C**), EDS of Cu-H-T@rGO (**D**), XRD of Cu-H-T, rGO, and Cu-H-T@rGO (**E**).

The morphology characteristics of the Cu-H, Cu-H-T and Cu-H-T@rGO were characterized by field emission scanning electron microscopy (FESEM). As shown in Figure 2A, single ligand EC-MOF Cu-H crystals present as cuboid with good facets, smooth surfaces, straight edges, and symmetrical structures. As shown in Figure 2B, double ligand EC-MOF Cu-H-T showed a smaller porous structure of particles. This may have a larger specific surface area and more exposure to BPA, indicating that Cu-H-T may provide more catalytic sites for the detection of BPA. Figure 2C shows that, after assembly with rGO in the proper proportion, the Cu-H-T nanoparticles uniformly grew on the surface and the intervals of rGO layers, indicating that rGO functions as a support and binder for nanocrystal growth. Notably, the regular interconnected Cu-H-T nanoparticles are distributed throughout the carbon framework, facilitating the processes of mass transport and charge delivery. The channel provides conditions for excellent performance during BPA detection. In Figure 2D, the EDS elemental map displays the existence of C, O, and Cu in Cu-H-T@rGO, which further confirms the FESEM results.

The crystallization and phase purity of different materials are further analyzed by powder X-ray diffraction (XRD). The XRD map of Cu-H-T, rGO and Cu-H-T@rGO is shown in Figure 2E. The XRD pattern of the Cu-H-T shows its low degree characteristic peaks at 9°, 13°, and 27°, corresponding to the (100), (130), and (002) planes, respectively. This is similar to Cu-HHTP in the literature [11,43]. In comparison, the synthetic composite Cu-H-T@rGO has the same peak level (star notation) as Cu-H-T and rGO, so it has a similar crystal structure, which indirectly confirms that the composite Cu-H-T@rGO has an MOF structure. All this indicates that the Cu-H-T@rGO has a similar crystal structure to Cu-H-T and has good crystallinity.

### 3.3. Electrochemical Characterization

**Figure 3:**
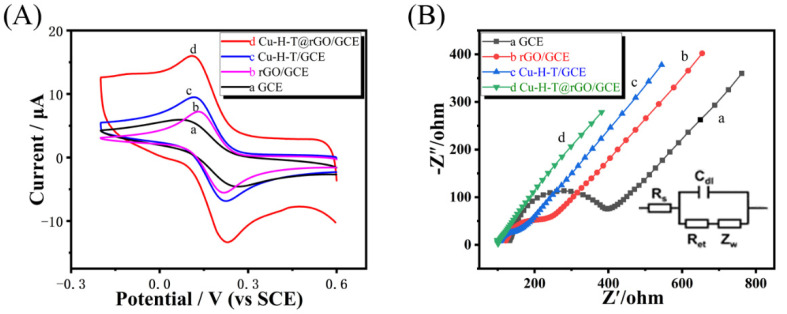
(**A**) CV and (**B**) Nyquist plots of bare GCE, rGO/GCE, Cu-H-T/GCE, and Cu-H-T@rGO/GCE recorded in 5 mmol·L^−1^ [Fe(CN)_6_]^3−/4−^ containing 0.1 mol·L^−1^ of KCl. The insert in (**B**) shows an equivalent circuit diagram applied to fit the Nyquist plots quantitatively.

The CV of bare (unmodified) GCE showed a pair of well-separated redox peaks (see Figure 3A), which indicates the reversible electrochemical reaction of [Fe(CN)_6_]^3−/4−^ on its surface. Redox peaks of rGO/GCE were slightly shifted and slightly higher than of the unmodified GCE. In Figure 3A, the highest current was observed for the GCE modified with double-ligand Cu-H-T and rGO (Cu-H-T@rGO/GCE. Thus, CV data confirmed the superior electrochemical performance of Cu-H-T@rGO/GCE relative to other modified GCEs tested in this work because Cu-H-T and rGO combination provided high surface area and conductivity, needed to adsorb reactive species and transfer the corresponding charges.

EIS tests showed that Cu-H-T@rGO/GCE possessed the lowest values of the charge transfer resistance (*R*_ct_) out of electrodes tested in this work (see Figure 3B), which is indicative of its ability to provide the fastest oxidation kinetics at the tight Cu-H-T/rGO interface.

### 3.4. Optimization of the Experimental Parameters

**Figure 4:**
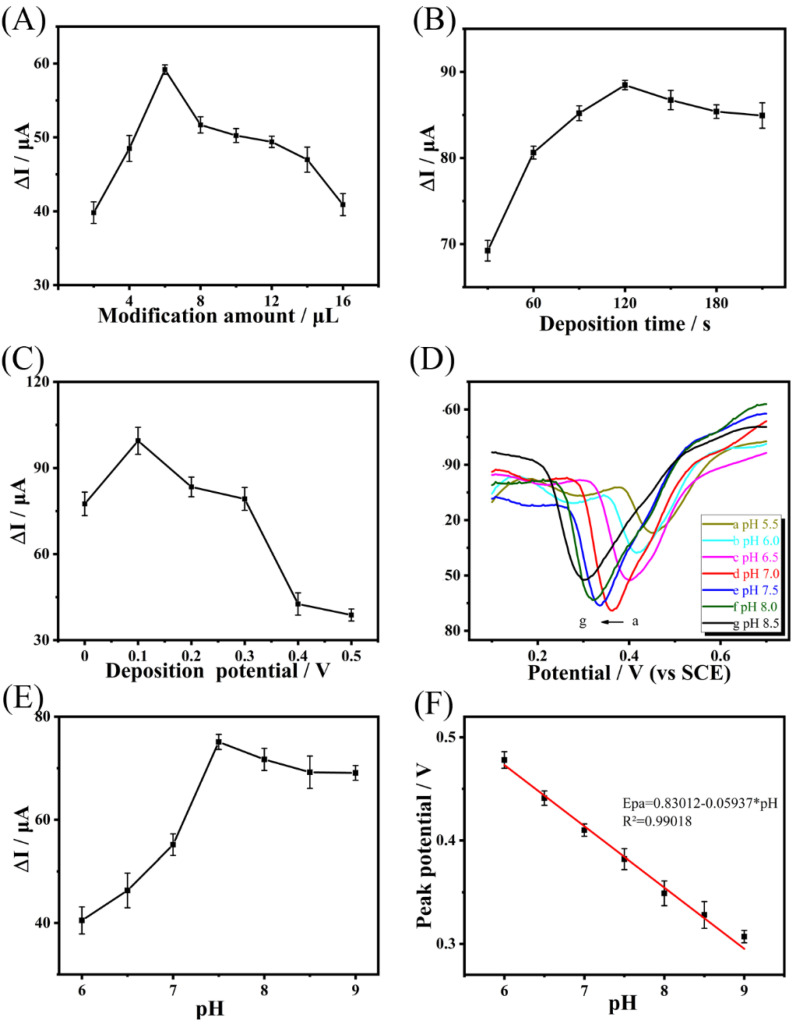
Current responses of the Cu-H-T@rGO/GCE-based electrodes to 0.1 mmol·L^−1^ BPA as a function of: (**A**) Cu-H-T@rGO amount on GCE, (**B**) GCE deposition time, (**C**) deposition potential, (**D**) DPV of Cu-H-T@rGO/GCE under the presence of 0.1 mmol·L^−1^ BPA at different pH values, changing from 5.5, followed by 6.0, 6.5, 7.0, 7.5, 8.0, to 8.5. Oxidation peak current (**E**) and potential (**F**) values plotted as a function of the corresponding pH values. The data were obtained in 0.2 mol·L^−1^ PBS. All data points in (**A**–**F**) are averages of five measurements, with the errors representing the corresponding standard deviations.

First, we tested how the amount of the Cu-H-T@rGO applied to GCE (which was in the 2–16 μL range) affected its electrochemical response to BPA. Figure 4A shows that, as the Cu-H-T@rGO nanocomposite volume was increased from 2 to 6μL, the peak oxide current values increased but then decreased very likely because an excessive and too thick Cu-H-T@rGO layer on GCE was blocking the electron exchange between BPA and the electrode [44]. Thus, 6 μL was chosen as the optimum amount to modify GCE. 

The deposition time and potential are also critical parameters affecting BPA detection by Cu-H-T@rGO/GCE. Thus, to optimize the sensitivity and detection limit of our Cu-H-T@rGO/GCE, we analyzed BPA adsorption/detection as a function of deposition time and potential (see Figure 4B). The peak oxidation current of the Cu-H-T@rGO/GCE electrode under the BPA presence increased as the cumulative time was changed from 30 to 120 s and remained stable up to 180 s. The peak oxide current of the value of Cu-H-T@rGO/GCE under the BPA presence increased only up to 0.1 V, after which it decreased, which indicates that smaller BPA amounts were absorbing on the Cu-H-T@rGO/GCE surface (Figure 4C). Thus, the best time to allow the Cu-H-T@rGO/GCE electrode to collect data before reading its final data was 120 s, and the best potential value for the electrochemical detection of BPA was 0.1 V.

The electrochemical reaction of BPA involves protons. Thus, BPA adsorption and electrochemical detection were expected to be pH-dependent. When the pH of the BPA solution in PBS was adjusted from 5.5 to 8.5 (Figure 4D), the peak current value increased but then decreased at pH above 7.0 (see Figure 4E). This optimized value (pH = 7.0) was below the pKa of BPA (equal to 9.73). Thus, BPA in its original form (with all protons still present) was adsorbed better on the Cu-H-T@rGO/GCE surface. In addition, the peak potential became negatively shifted as the pH of the BPA solution was increased (see Figure 4F), confirming that protons were directly involved in its electrochemical oxidation. *Epa* values plotted as a function of pH showed linear dependency with the correlation coefficient R^2^ = 0.99018 and a curve described as *Epa*(V) = 0.83012 − 0.05937·pH. Thus, the slope value, equal to 59.10 mV, is very close to the theoretical Nernst value for this process [45]. Thus, the electrochemical oxidation of BPA on the Cu-H-T@rGO/GCE surface occurred with an equal number of electrons and protons.

### 3.5. Effect of Scan Rate

**Figure 5:**
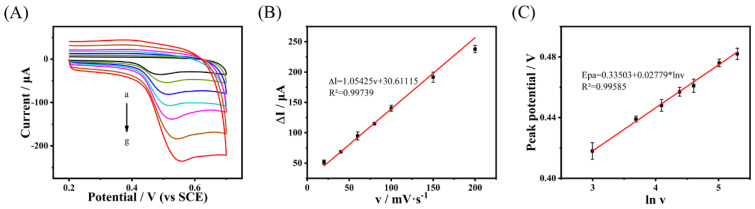
(**A**) CV of the Cu-H-T@rGO/GCE under the presence of 0.1 mmol·L^−1^ BPA solution (in 0.2 mol·L^−1^ PBS with pH = 7.0) at 20, 40, 60, 80, 100, 150 and 200 mV·s^−1^ scan rates (marked as a→g). (**B**) The oxidation peak current and (**C**) peak potential as functions of scan rates (**B**) and their natural logarithms (**C**). All data points in (**B**,**C**) represent an average value of five measurements with the errors calculated as standard deviations.

BPA oxidation mechanism on the Cu-H-T@rGO/GCE surface was studied by CV at different scan rates (see Figure 5A). The BPA peak oxidation current value increased linearly with the scan rates (see Figure 5B) with the corresponding linear regression equation expressed as: Δ*I* = 1.05425·*v* + 30.61115 (R^2^ = 0.99739). Thus, the electrochemical BPA oxidation on the Cu-H-T@rGO/GCE surface was a typical adsorption-control process. This data confirmed that Cu-H-T@rGO was an excellent choice to modify GCE for BPA detection. Additionally, the peak oxidation potential also increased as the scan rate was increased (see Figure 5C). The relationship between the peak potential (*Epa*) and the natural logarithm of the scan speed in the 20–200 mV∙s^−1^ range could be described by the following linear expression: *Epa* = 0.33503 + 0.02779·ln*v* (R^2^ = 0.99585). 

**Scheme 2:**
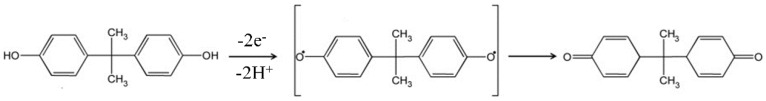
Oxidation mechanism of BPA at Cu-H-T@rGO/GCE.

Typically, the relationship between potential (*Epa*) and scan rate (*v*) for the adsorption-controlled and irreversible electrode processes could be expressed using Laviron’s Law [46]:

*Epa* = E0′ + (RT/*αn*F)ln(RTks/*αn*F) + (RT/*αn*F)lnv

where E0′ is the apparent potential, ks is the electrochemical rate constant, n is the number of transferred electrons, α is the transfer coefficient, T is the temperature (equal to 298 K), F is the Faraday constant (equal to 96,487 C·mol^−1^), and R is the gas constant (equal to 8.314 J·mol^−1^·K). The *αn* value could be obtained from the slope of the upper curve (0.0265). Thus, *αn* is equal to 0.99. For a completely irreversible electrochemical process, the value of α is assumed to be 0.5 [47]. Therefore, as shown in Scheme 2, the number of electrons transferred during BPA oxidation is equal to 2, which confirms that BPA oxidation on the Cu-H-T@rGO/GCE surface was adsorption-driven dual-electron and dual proton reaction.

### 3.6. Analytical Performance

**Figure 6:**
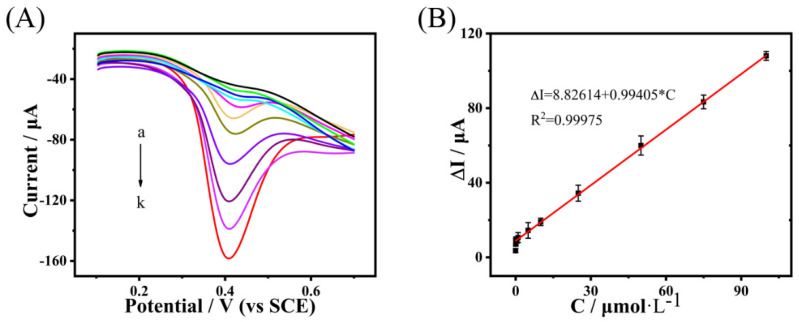
(**A**) DPV curves recorded for the Cu-H-T@rGO/GCE electrode under the different (0.01, 0.05, 0.1, 0.5, 1, 5, 10, 25, 50, 75, and 100 μmol·L^−1^, marked as a→k) BPA solutions in 0.2 mol·L^−1^ PBS (with pH = 7.0); (**B**) oxidation peak current plotted as a function of the BPA concentration.

After determining the optimized Cu-H-T@rGO/GCE-based sensor, its BPA detection performance was studied by DPV (see Figure 6A). The peak current of the Cu-H-T@rGO/GCE increased linearly (with the correlation coefficient R^2^ = 0.99975) with the BPA concentration (see Figure 6B). This dependence could be expressed as Δ*I*(μA) = 8.82614 + 0.99405·*C*, where *C* is BPA concentration (in μmol·L^−^^1^). The detection limit calculated from this equation was established to be equal to 3.6 nmol·L^−1^ (S/N = 3). 

The performance of our novel BPA electrochemical sensors based on the Cu-H-T@rGO/GCE was compared with other similar systems reported in the literature (see Table 1). Compared to the previously fabricated sensors, our novel and easy-to-prepare Cu-H-T@rGO/GCE-based sensor was excellent and very sensitive towards BPA detection.

### 3.7. Reproducibility, Stability and Interference Performance of the Cu-H-T@rGO/GCE Sensor during BPA Detection 

Reproducibility, stability, and performance under the interference of other chemicals are the essential parameters determining the practical applications of a sensor. Thus, all these parameters were tested with regard to our novel Cu-H-T@rGO/GCE sensor. The reproducibility of the same Cu-H-T@rGO/GCE electrode during the BPA detection was excellent, judging by the 1.5% deviation for five consequent BPA detection measurements. Such performance was significantly better than other BPA sensors [48,52]. The long-term stability of our Cu-H-T@rGO/GCE-based sensor was assessed by testing it against BPA presence two times per week during the five-week storage at room temperature. The Cu-H-T@rGO/GCE-based sensor response at the fifth measurement was 93% of its initial response, which indicates its excellent storage stability.

The tests to determine the influence of the presence of potentially interfering compounds on the Cu-H-T@rGO/GCE performance during the BPA detection were also conducted (see Table 2). For this purpose, we added several chemicals (Na^+^, Mg^2+^, K^+^, NH_4_^+^, NO^3−^, Cl^−^, CO_3_^2−^, and SO_4_^2−^**)** to 0.1 mmol·L^−1^ BPA solution. Even at the concentrations of these interfering compounds exceeding the BPA contents 100 times, the BPA oxidation peak observed for our Cu-H-T@rGO/GCE-based sensor did not change. When other organic chemicals were added (such as POP, PNP, and TBBPA) at concentrations exceeding BPA content by five times, the BPA was still detected with excellent accuracy and RSD of only 4.6%. Thus, there was minimum interference from these compounds during BPA detection by our novel Cu-H-T@rGO/GCE-based sensor.

### 3.8. Practical Applications of the BPA Detection by the Cu-H-T@rGO/GCE

Frequently, the best way to analyze the influence of the interfering chemicals, especially during the analyte detection in real-life samples and in complex matrices, is to use an addition method. Therefore, for this purpose, we applied our Cu-H-T@rGO/GCE-based sensor to detect BPA in real water samples using the standard DPV-based procedure. We purchased two plastic bottles from a local supermarket, filled them with PBS, and placed them in the sun for a week to induce BPA from the plastic. The resulting solution was filtered by a 0.22 μm membrane and then analyzed by our Cu-H-T@rGO/GCE-based sensor using DPV. We detected no BPA in the water (see Table 3). Therefore, we then used a standard addition method and spiked the samples up to 0.80, 3.00, and 50 μmol·L^−1^ of BPA. DPV analysis performed with our electrode detected 94.6–104.3% of the added BPA content (see Table 3). Thus, our Cu-H-T@rGO/GCE-based was accurate in detecting BPA in real water samples.

## 4. Conclusions

This work reports the preparation of a series of double-ligand EC-MOFs materials (M-H-T). After several screening tests, conductive Cu-H-T was selected to prepare a modified Cu-H-T@rGO/GCE, which was then used for rapid, accurate, and sensitive detection of BPA in the 0.05–100 μmol·L^−1^ range and 3.6 nmol·L^−1^ detection limit. We also used this method to determine BPA in plastic beverage bottles directly and also by the addition method. Our sensor was able to detect BPA levels with recoveries ranging from 94.6% to 104.3% (RSD ≤ 4.5). This facile method introduces a novel strategy based on the double-ligand EC-MOFs Cu-H-T grown on rGO for efficient BPA determination, which offers a novel opportunity for electronic conductive MOFs as electro-catalysis materials in the practical application.

## Figures and Tables

**Table 1:** Comparison of the performances of the BPA sensors fabricated in the literature and this work.

Modified Electrode	Method	Linear Range (mol·L^−1^)	LOD (nmol·L^−1^)	Reference
AuNP/SGrNF/GCE	LSV	8.0 × 10^−8^–2.5 × 10^−4^	35.0	[48]
PANINR/MWCNT/PGE	Amp	1.0 × 10^−6^–4.0 × 10^−4^	10.0	[49]
NiO/CNT/IL/CPE	SWV	8.0 × 10^−8^–5.0 × 10^−4^	40.0	[50]
SWCNT/PEDOT/GCE	CV	1.0 × 10^−7^–5.8 × 10^−6^	32.0	[51]
AuNP/Cu-MOF/CPE	DPV	2.0 × 10^−4^–1.0 × 10^−3^	37.8	[31]
Ce-Ni-MOF/MWCNTs/GCE	DPV	1.0 × 10^−7^–1.0 × 10^−4^	7.8	[32]
Cu-H-T@rGO/GCE	DPV	5.0 × 10^−8^–1.0 × 10^−4^	5.2	This work

**Table 2:** Maximum tolerable concentration of coexisting substances with 0.1 mmol·L^−1^ BPA.

Coexisting Substances	Tolerance Limit (mmol·L^−1^)	Relative Deviation (%)
NaNO_3_	10	1.8
MgCl_2_	10	2.1
K_2_CO_3_	10	1.6
(NH_4_)_2_SO_4_	10	2.7
POP	0.5	4.6
PNP	0.5	2.3
TBBPA	0.5	3.8

**Table 3:** BPA detection by the Cu-H-T@rGO/GCE in the real samples.

Sample	Measured (μmol·L^−1^)	Added (μmol·L^−1^)	Found (μmol·L^−1^)	RSD (%)	Recovery (%)
Beverage bottle Ⅰ	0.00	0.80	0.78	2.3	97.5
		3.00	2.96	3.1	98.6
		50.00	51.07	4.5	102.1
Beverage bottle Ⅱ	0.00	0.80	0.75	0.8	93.8
		3.00	3.13	1.7	104.3
		50.00	48.55	4.2	97.1

## Data Availability

Data may be obtained via email to ruihye@163.com or huangdihui@163.com.
